# Attitude to Human Papillomavirus Deoxyribonucleic Acid-Based Cervical Cancer Screening in Antenatal Care in Nigeria: A Qualitative Study

**DOI:** 10.3389/fpubh.2017.00226

**Published:** 2017-09-06

**Authors:** Temitope E. Filade, Eileen O. Dareng, Toyosi Olawande, Tolani A. Fagbohun, Amos O. Adebayo, Clement A. Adebamowo

**Affiliations:** ^1^Institute of Human Virology, Abuja, Nigeria; ^2^Department of Public Health and Primary Care, University of Cambridge, Cambridge, United Kingdom; ^3^Department of Obstetrics and Gynaecology, Asokoro District Hospital, Abuja, Nigeria; ^4^Institute of Human Virology and Greenebaum Comprehensive Cancer Centre, University of Maryland School of Medicine, Baltimore, MD, United States; ^5^Department of Epidemiology and Public Health, University of Maryland School of Medicine, Baltimore, MD, United States

**Keywords:** human papillomavirus, HPV DNA testing, cervical cancer, cervical cancer screening, antenatal care, pregnant women, pregnancy

## Abstract

**Objectives:**

Human papillomavirus (HPV) deoxyribonucleic acid (DNA)-based testing is increasingly used for cervical cancer screening in developed countries, but the best approach to utilizing it in low- and middle-income countries (LMIC) is unclear. Incorporation of HPV DNA-based testing into routine antenatal care (ANC) is a potential yet poorly explored strategy for cervical cancer screening in LMIC. We explored the attitude of health care workers and pregnant women to the incorporation of HPV DNA-based tests into routine ANC in Nigeria.

**Methods:**

We conducted nine focus group discussions with 82 pregnant women and 13 in-depth interviews with obstetricians and midwives at four health care facilities in Abuja, Nigeria. We used qualitative content analysis to analyze the data and the theory of planned behavior as a theoretical framework to examine the responses.

**Results:**

Pregnant women expressed a favorable attitude toward HPV DNA testing for cervical cancer screening as part of routine ANC. Acceptability of this approach was motivated by the expected benefits from early detection and treatment of cervical cancer. The factors most commonly cited as likely to influence acceptability and uptake of HPV DNA-based tests are recommendations by their care providers and mandating testing as part of ANC services. Discussants mentioned lack of awareness and affordability as factors that may inhibit uptake of HPV DNA-based cervical cancer screening. Midwives expressed concerns about the safety of sampling procedure in pregnancy, while obstetricians fully support the integration of HPV DNA-based testing into routine ANC.

**Conclusion:**

Our results show that incorporating HPV DN-based cervical cancer screening into routine ANC is acceptable to pregnant women and health care providers. Making the test affordable and educating health care workers on its efficacy and safety if performed during ANC will enhance their willingness to recommend it and increase its uptake.

## Introduction

Cervical cancer is the second commonest cancer among women and the third leading cause of cancer deaths in low- and middle-income countries (LMIC) (1–3). Of the estimated 528,000 new cases that occurred globally in 2012, around 85% occurred in LMIC (1). The incidence of cervical cancer in developed countries has fallen significantly over the past five decades due to widespread implementation of screening programs (4). However, the screening technologies have not been successfully transferred to LMIC and, in these countries, cervical screening has generally been ineffective in reducing cervical cancer mortality. In a household survey of cervical cancer screening coverage conducted as part of the World Health Survey, Gakidou et al. reported coverage ranging from 1% to 23% in African countries (5). In contrast, screening coverage in European countries ranged from 51 to 82% (5). In Nigeria, 50.3 million women were considered to be at risk for cervical cancer in 2015 and the estimated cervical cancer screening coverage in the general population ranged from 1.4 to 8.7% (6).

Several methods of conducting cervical cancer screening in LMIC have been evaluated. These include visual inspection with acetic acid (VIA) or Lugol’s iodine (VILI) and human papillomavirus (HPV) deoxyribonucleic acid (DNA)-based testing performed on either self-collected or health care provider (HCP)-collected cervico-vaginal samples (7). While touted for their simplicity, ease of deployment and low cost, VIA, and VILI have not been associated with reductions in cervical cancer mortality (8). VIA/VILI are also highly subjective and have low sensitivity and specificity leading to frequent false positives and overtreatment. Studies of the feasibility and acceptability of HPV DNA-based testing have shown that HPV DNA-based tests are acceptable and feasible for cervical cancer screening in LMIC (9–12). Reasons include its potential for high-throughput, greater objectivity, ability to provide long-term risk stratification and higher sensitivity (13). The latter is particularly important in LMIC where women are likely to be screened only once or twice in their lifetimes because of lack of resources and poor follow-up culture. Other benefits of HPV DNA-based tests include possibility of using self-sampling, fewer clinic visits, and reduced infrastructural requirements compared to other methods, e.g., Pap smear, which requires well-trained cytotechnologists that are in limited supply in LMIC (13–16).

One method for increasing cervical cancer screening coverage is its integration into existing and popular women’s health programs such as routine antenatal care (ANC)—the umbrella program that delivers a range of treatment and prevention services to pregnant women (17–19). Studies have shown that a high proportion of women in LMIC have at least one ANC visit during their pregnancy. The prevalence of these visits ranges from 84.6% in Nigeria to 97% in Tanzania (20–24). There is also evidence that women attending ANC demonstrate a strong motivation to adhere to medical follow-up and care instructions throughout the course of pregnancy (17). Public health interventions, such as human immunodeficiency virus (HIV) testing and malaria control with insecticide-treated bed nets, have been delivered through ANC with notable degree of success (25, 26). Though a few studies have evaluated the feasibility and safety of the use of cytology-based screening and HPV DNA-based screening in pregnancy (18, 27, 28), none has compared the acceptability of various cervical cancer screening methods as part of routine ANC. In this study, we investigate the acceptability of integrating cervical cancer screening using HPV DNA-based tests into routine ANC among Nigerian women and health care providers (HCP), as an innovative approach to increasing cervical cancer screening coverage.

## Materials and Methods

### Study Design and Setting

We applied the theory of planned behavior (TPB) and an interpretivist approach to gain an understanding of the perceptions and willingness of pregnant women and HCP to accepting the incorporation of HPV DNA-based tests for cervical cancer screening into routine ANC in Nigeria. According to the TPB, behavioral intention is influenced by one’s attitude toward performing a behavior, beliefs about whether individuals who are important to the person approve or disapprove of the behavior (subjective norm) and perceived behavioral control, which has to do with one’s belief that they can control a particular behavior (29–30). For this study, “behavioral intention” is defined as the willingness of women to screen for cervical cancer during ANC using HPV DNA-based test. We conducted nine focus group discussions (FGD) among 82 pregnant women between June 2015 and April 2016 at Asokoro District Hospital and Kuchingoro Maternal and Child Health Facility, Garki Hospital, and Nisa Premiere Hospital, all of which are located in Abuja, central Nigeria. We also conducted 13 in-depth interviews with HCP at these health institutions.

### Study Participants

#### Pregnant Women

We hypothesized that acceptability of HPV DNA-based cervical screening during ANC may be influenced by religious beliefs; therefore, we enrolled pregnant women from the two major religious groups in Nigeria—Christian and Muslim (31, 32). We conducted group discussions in both rural and urban areas of Abuja. We determined sample size based on an emergent theory approach, which utilizes the saturation sampling model. In this model, sample size is determined by a process in which interviews are conducted sequentially, until the point of data saturation or redundancy (33). We used the purposive sampling approach to identify hospitals and health care centers in both rural and urban areas where we can recruit participants (34). Pregnant women were eligible if they were registered to attend ANC in health care facilities rather than with traditional birth attendants, were resident in Abuja, and had no previous history of cervical cancer. We provided the women with information about the study and asked if they would be interested in participating. All the women who were approached accepted the invitation, and we provided them with information on the date, time, and venue for the FGD.

#### Health Care Providers

We approached obstetrics and gynecology (O&G) consultants, resident doctors in O&G departments, midwives, and community health officers, in the four health facilities selected for this study and invited them to participate in in-depth interviews. HCP were eligible to participate if they were involved in providing ANC services in any one of the five health facilities selected for this study. We selected potential interviewees purposively until we reached data saturation and considered that additional interviewees were not contributing new information. Individual interviews were scheduled at a convenient venue and time for each participant. Data collection from the interviews ended when data saturation were attained.

### Study Procedures

#### Focus Group Discussions

We developed interview guides based on our research objective, carefully wording the questions to avoid pre-loading the guide with any concepts. In brief, our interview guide was divided into three sections: knowledge of cervical cancer screening, attitude toward HPV DNA-based cervical cancer screening in pregnancy, and practice of HPV DNA-based cervical cancer screening as part of routine ANC. Our opening questions were on knowledge of cervical cancer and were designed to be non-sensitive to get participants comfortable. HPV DNA-based testing was explained as one of the methods for cervical cancer screening. We pre-tested the interview guide with a group of six women and revised it according to feedback from our pre-test participants. TEF (a female nurse/research scientist) and TAF (a female nurse/research scientist) moderated all the FGDs. Both TEF and TAF are highly skilled and experienced in the conduct of qualitative interviews and together have up to 3 years’ experience in conducting qualitative studies. All FGDs were conducted in English language, in enclosed rooms. A competent interpreter was present to translate questions and responses in one of the focus group sessions. Each FGD lasted between 45 min and 1 hr. All sessions were audio-recorded, and supplementary descriptive notes were taken during each session. We transcribed each audio recording verbatim. Transcripts and descriptive notes were used for analysis.

#### Key Informant Interviews (KII)

We used a semi-structured interview guide to conduct in-depth interviews with seven obstetricians, five midwives, and one community health extension worker. In brief, the interview guided included three sections: opinions about current cervical cancer prevention strategies and screening uptake; attitudes toward HPV DNA-based cervical cancer screening as part of routine ANC; and perceived adverse events associated with the introduction of HPV DNA-based cervical cancer screening into routine ANC. Our opening questions on opinions about current cervical cancer screening prevention strategies in Nigeria were designed to get participants comfortable. HPV DNA-based testing as a method of cervical cancer screening was explained as part of the introduction and rationale of the study. Each interview session lasted between 30 and 45 min and was conducted in private rooms. All interviews were facilitated by TEF and conducted in English language.

### Data Analysis

We used qualitative methods to analyze the FGD and KII transcripts with a combination of deductive and inductive approaches using Atlas.ti^®^ version 7.5.10. Temitope E. Filade, Eileen O. Dareng, and Toyosi Olawande developed the coding frame through an iterative process. We used a concept driven approach to create *a priori* codes based on the research objective as a general framework, while allowing emergent themes from the transcripts and field notes. We evaluated the coding frame for unidimensionality, mutual exclusiveness, and exhaustiveness by coding one FGD transcript and one in-depth interview transcript and reviewing the results at a data analysis review meeting. Temitope E. Filade and Toyosi Olawande used the agreed-upon coding frame to code all transcripts. This round of coding yielded descriptive codes. Next, we applied the TPB to review and interpret the descriptive codes yielding more analytical codes. Transcripts and results were not returned to participants for comments.

### Ethical Considerations

The study was approved by the National Health Research Ethics Committee of Nigeria (NHREC), and we obtained written informed consent from all participants.

## Results

### FGD with Pregnant Women

Some 82 pregnant women participated in the nine FGDs. The mean (SD) age of the participants was 28.9 (4.7) years. Most were Christians (63.4%), 95.1% had some years of formal education, 53.7% were employed, and all were married. None of the participants had ever been screened for cervical cancer.

#### Knowledge

Knowledge of cervical cancer, its symptoms and risk factors were limited across all FGD participants. Despite the limited knowledge overall, Christian and urban dwelling participants were better informed about cervical cancer and its symptoms than Muslim and rural dwelling participants. Of the more knowledgeable Christian women, most attributed cervical cancer to sexual practices like early coitarche and having multiple sexual partners. The less well-informed women either did not know any risk factor for cervical cancer or believed miscarriages and use of cosmetics were risk factors.

“Having multiple sex partners is very very risky. I think in one way or the other it contribute to let me say growth or development of cervical cancer.” (Christian participant 4, group I, urban dweller)

“It’s caused by Human Papilloma Virus through sex.” (Christian participant 3, group II, urban dweller)

“I don’t know the cause. I’m just hearing about cancer here.” (Muslim participant 7, group II, rural dweller)

“I think it’s too much of miscarriage. It can cause cervical cancer.” (Muslim participant 4, group II, rural dweller)

“All these skin bleaching something, I mean, cream that bleaches body, they’re also causes of cancer.” (Muslim participant 2, group I urban dweller)

Because the FGDs were interactive, participants who had initially indicated that they were unaware of cervical cancer were able to participate in the discussions once the more aware participants used local terminologies such as “cancer of the neck of the womb” and “cancer of the birth canal” to describe cervical cancer.

#### Attitude

The major themes identified in the FGDs are summarized in Table 1. Though most of the participants had poor knowledge of cervical cancer, they had a favorable attitude toward the use of HPV DNA-based tests for cervical cancer screening during routine ANC. Most participants in the FGDs believed routine HPV DNA screening would be “good” or “helpful” because cervical cancer could be detected early:

“…it [HPV DNA based testing] will help if it is detected early, so that one can have treatment for that [i.e. cervical cancer]” (Christian participant 5, group I, urban dweller)

“It [HPV DNA based testing] should be a routine test because uhm, it will make the disease less common, because in the United States, it’s normal, just like this HIV stuff. It’s normal there so, the rate is not as the African continent.” (Christian participant 3, group IX, urban dweller)

“We will do it [HPV DNA based testing] but what we need most is knowledge on it. You know when they said you go and do HIV test, everybody knows what HIV is, you know what they’re looking for, you know the cause. So once we know what we suppose to know about this cervical cancer screening, once they said you need to do it, there will be no fear.” (Muslim participant 5, group IV, urban dweller)

**Table 1:** Major themes identified based on the tenets of the theory of planned behavior.

Tenets of theory of planned behavior		Pregnant women (focus group discussion)	Health care provider (in-depth interview)
Attitude	Overall perception of human papillomavirus deoxyribonucleic acid-based screening in pregnancy	Favorable	Favorable Avoid high-risk pregnancies

	Beliefs about consequences	Increased susceptibility to cervical cancer during pregnancy Early detection is beneficial Concerns about the safety of the baby	Increased cervical cancer screening coverage Concerns about safety of screening procedure

	Outcome evaluations	Better rates of survival/cure with early detection Increased anxiety with early detection	Increased cervical cancer screening coverage

Subjective norms	Normative beliefs	Health care provider Spouse Other pregnant women	None identified

	Motivation to comply	Health care providers know what is best for their patient Health care providers would not recommend harmful practices Experiences, adverse events from other women who have gone through the screening Mandatory antenatal care policy	

Perceived behavioral control	Capability	Increasing awareness among pregnant women	Awareness Religious and cultural beliefs Include robust counseling arm

	Control	Mandatory ANC screening Cost Availability Religious beliefs	Cost Availability

*Attitude: refers to the overall evaluation of HPV DNA-based cervical cancer screening as part of routine ANC. It involves the two components: belief about consequences and corresponding negative or positive judgment about these consequences (outcome evaluation)*.

*Subjective norms: This is an evaluation of the social pressures to participate in HPV DNA-based cervical cancer during pregnancy as part of routine ANC (normative belief) and motivations to comply with these pressures (motivation to comply)*.

*Perceived behavioral control: the women’s belief that they can participate in HPV DNA-based cervical cancer screening during pregnancy (capability) and factors that may inhibit or facilitate this behavior (control)*.

*HPV, human papillomavirus; DNA, deoxyribonucleic acid; ANC, antenatal care; FGD, focus group discussion*.

Majority of the participants in the FGD believed that they were not only at increased risk of diseases during pregnancy but that existing infections and abnormalities of the cervix may be exacerbated by pregnancy. They were therefore happy to accept screening for cervical cancer using HPV DNA-based methods during pregnancy.

“Yes I think a pregnant woman is at risk. Why I said a pregnant woman is at the risk of developing it [cervical cancer]; is that when a woman is pregnant, we’re talking about cervix and the whole thing happens around there. Cervix encloses the womb. So I think there’s a risk there and when a woman is pregnant, immunity is low. So I think if there’s any trace of cervical cancer at that moment, already existing around there, when a woman is pregnant, I think that is when it will even manifest the more because there’s a lot of reactions going on around there.” (Christian participant 3, group II, urban dweller)

While several participants believed that early detection of cervical cancer using HPV DNA-based testing was beneficial because it would lead to better treatment and a higher chance of excellent outcomes, some participants opined that anxiety about results of screening tests would be a deterrent for them. Other deterrents to screening using a HPV DNA-based method during the antenatal period mentioned by participants were distrust of the screening process, risk of acquiring new infections, and the safety of the screening procedure during pregnancy. Some of the women stated that they would consent only if they are convinced of the safety of the procedure for their unborn baby.

“It [HPV DNA based testing] might be dangerous for the baby inside because we don’t know how powerful the test [HPV DNA based testing] is, we don’t know the kind of machines they use…if it is something like x-ray, they said x-ray is not good for pregnant women.” (Muslim participant 5, group IV, urban dweller)

#### Subjective Norms

Most women indicated that the single most important influence on their decision to participate in a cervical cancer screening program using HPV DNA-based methods during ANC would be the recommendation of their HCP. This was true regardless of gravidity, religious affiliation, or dwelling place. The strongest motivation for the women to comply with their HCP’s recommendation is belief that the HCP know what is best for the women’s health and would not recommend procedures that would be harmful to their babies.

“…if they (health care professionals) ask her to do the screening then that means it is okay doing it.” (Christian participant 4, group I, urban dweller)

Another influential factor mentioned by the discussants is the experience and opinion of other pregnant women who undergo the screening procedure. Most said that their decision to participate would be based to a large extent on other pregnant women’s report of a pleasant screening experience; this may help to assuage any fears that they may have about HPV DNA-based cervical screening procedure.

“Like now, if I first do, I go fit tell them say, den say den see sickness, den see that sickness for me, or den no see that sickness for me. Maybe den go say den want to do and see maybe den get am too.” [For instance, I could go in to do the test first, then share my experience with other pregnant women, maybe they could be convinced to go for it] (Muslim participant 7, group VI, rural dweller)

An additional theme identified in all FGDs is the introduction of mandatory HPV DNA testing for cervical cancer screening with an “opt out” strategy as part of routine ANC programs similar to how HIV testing is currently done in Nigeria. Participants opined that with this approach, all women would be offered HPV DNA based cervical cancer screening at their ANC appointments, except those who specifically opt out or are judged to have high-risk pregnancies by their obstetricians.

“I think that’s the only way you can really bring in women to do it [HPV DNA based cervical cancer screening], being they include it among antenatal…because if you come for antenatal now, definitely whether you like it or not you must take part in it, like urine test. But say on a more neutral note, it’s not inclusive, even after antenatal; we’ll still go back home and forget about it.” (Christian participant 5, group II, urban dweller)

Participants further opined that adequate education and awareness creation about the need for HPV DNA-based cervical cancer screening would provide a strong motivation to comply with such a screening policy.

#### Perceived Behavioral Control

None of the pregnant women in this study had ever been screened for cervical cancer. The commonest reasons for this included lack of awareness and lack of available screening facilities. These women affirmed that if they were enlightened on the availability of HPV DNA-based tests and other methods for cervical cancer screening, they would screen for cervical cancer. They further stated that health workers have the responsibility of educating them on cervical cancer screening to help their decision-making.

“If you didn’t tell me what cervical cancer is all about, the risk in the test [HPV DNA testing], how it will affect me or the baby, I will hesitate. But if you give me a pre-knowledge about it like we all know HIV, we all know why we take HIV test as a pregnant woman, so one will not hesitate.” But if I don’t know anything about cervical cancer, I will hesitate.” (Christian participant 7, group II, urban dweller)

“It’s not every hospital they use to do it [HPV DNA based testing for cervical cancer screening]. It’s not all hospitals except the hospital that have equipments. The test is not common like AIDS or those things. They have specific hospitals for it. So like primary health center now, you can’t see it there.” (Muslim participant 6, group III, urban dweller)

In addition, FGD participants mentioned potential for early detection of the disease, presence of early signs and symptoms, and desire for a successful pregnancy as other compelling factors that would influence their compliance with an HPV-based DNA cervical cancer screening program.

“If you don’t have any sickness in your body, and you don’t have anything in your body, you cannot take yourself to hospital to go and find out. Unless when you have something in your body that you go to hospital to find out, is from that place that they will get it, they will know what is wrong with you.” (Muslim participant 3, group VIII, urban)

Other factors that the participants mentioned which would affect their decisions on uptake of HPV DNA-based cervical cancer screening included cost of screening, procedure-associated adverse events, spouse’s opinion, fear of screening outcome, and fear of the screening equipment/procedure.

“Some may ask whether any payment is involved. Finance can also determine one’s decision. The person may say I’m spending a lot, I’m pregnant, I’m buying things, since money is involved let me wait, when I’m through with the delivery, I can now prepare and come for it.” (Christian participant 3, group II, urban dweller)

“Some people are scared of knowing their status. So, they don’t want to know. They’ll be scared of knowing what is wrong with them, so that they will not have BP problems, start thinking, ‘ha, I have HIV, I have cancer’ that fear can make you not to go. You’ll just prefer to leave it; you’ll just say ‘let me leave it.’?” (Christian participant 9, group VIII, urban dweller)

Religious inclinations and beliefs did not appear to negatively affect the likelihood that our FGD participants would participate in HPV DNA testing for cervical cancer screening. Muslim women especially were convinced that Islamic religion pioneered and gives full support to participating in preventive health care activities, as seen in their pre-prayer washing—“ablution.”

“Islam is even the one that really explains what health, neatness, everything about health is…everything about health! So when they are saying something like this, that affects somebody, in fact, you’re free to go and do it.” (Muslim participant 5, group IV, urban dweller)

### In-Depth Interviews with Health Care Providers

The HCP interviewed in this study believed cervical cancer is a major public health challenge for Nigerian women and that uptake of cervical cancer screening is rather low. Several factors were believed to be responsible for this; the main one being “lack of awareness,” which leads to presentation at advanced stage of disease in hospitals.

“Well, my opinion about cervical cancer in Nigeria, is that it’s still remains a major problem, yeah. As the total awareness of the general population is still not adequate with regards prevention, so a lot of times, people don’t go for screening as recommended. And first time you’re seeing them, they may have a full-blown disease or its complication. The morbidities, mortalities or so, associated with it become a problem but it’s still very common, one of the commonest cancers affecting women. Then the fact that it’s the commonest genital cancer in this part of the world…in Nigeria particularly, so it remains a big problem, that’s the truth.” (KII 11, O&G)

Other reasons responsible for low screening uptake mentioned by the HCPs include high screening costs, poor availability of screening facilities, fear of the unknown, lack of awareness, and religious/cultural beliefs.

“With the economic situation, if you ask somebody to pay five hundred naira, one thousand naira, two thousand naira for something she’s not even aware of…people try to shy away. If we can bring down the cost, if possible free-of-charge, it will also help to increase the uptake.” (KII 4, O&G)

“As easy as the common Pap smear is, as easy as it can be done, it’s not in every center. In an entire state, you don’t have maybe more than two or three places. You may not know it if you’re coming from Lagos or some other places because you have a lot of people who are educated. Go down to the north, apart from where NGOs are sponsoring, you will have issues and it is a significant problem.” (KII 11, O&G)

The HCPs further stated that there is significant disparity in the availability of cervical cancer screening comparing rural and urban locations. They believed that most of the women in urban areas have better access to cervical screening services due to a higher level of exposure to health information in the city and availability of well-equipped health care facilities, as opposed to the rural areas.

“The large percentage of people living in Nigeria are those people that are living in the rural areas. So, people still need to be well informed in the local areas.” (KII 1, Midwife)

“Well, I think the coverage is more in the urban centers, the facilities are more in the urban centers. I will say in the rural areas, there’s some degree of neglect, neglect of our women you know, in terms of the facilities that are available for testing. So there’s more concentration of testing kits, or testing facilities, more in the urban center.” (KII 3, O&G)

The obstetricians especially believed that introducing HPV DNA-based cervical cancer screening into routine ANC will be an innovation aimed at improving cervical cancer screening uptake in the country. It will lead to positive outcomes if coupled with proper counseling and informed consent:

“It will be very good honestly because this is more of a higher technology. It’s better for us in terms of the sensitivity, because the Pap smear we do has up to ten to twenty per cent, the sensitivity is low. But this one is better. So I think at the end of the day, it will go a long way in helping…you know vis-a-vis changes in pregnancy.” (KII 11, O&G)

“Until now, HPV DNA testing has been non-existent or has been non-existent in this country and I’m sure your organization is one of the first to bring HPV DNA testing into routine use. It doesn’t have any implications on maternal or fetal health. It’s a non-invasive procedure. I don’t think it will cause any harm to the woman.” (KII 09, O&G)

“Yeah, it’s a good thing. Now to start with, in antenatal clinics you have more contacts with women there. So I think you can capture them, if at all, if there’s any form of early detection, it can be handled.” (KII 10, O&G)

Among HCP, only midwives were unfavorably disposed toward the introduction of HPV-based DNA screening into routine ANC. Their concerns were that the sample collection process for HPV DNA-based testing may be unsafe in pregnancy and there may be risk of loss of pregnancies with attendant emotional and legal consequences.

“One should be very very careful when a woman is pregnant to tamper with the cervix, because tampering with the cervix at a certain stage might bring about abortion. It’s better to make sure at a certain stage in life, women are encouraged to go for your cervical screening testing before you get married or before you start having your babies, not during pregnancy.” (KII 01, Midwife)

Most of the gynecologists did not believe that there would be any adverse events from obtaining cervical samples from pregnant women for HPV DNA-based screening; however, some thought that women with high-risk pregnancies should be excluded and re-scheduled for a post-partum appointment.

“Well, from my own professional point of view, women who have some challenges in pregnancy usually one may just avoid such women from carrying out the test. Women who have maybe abortion, who are bleeding…any form of bleeding during pregnancy, high-risk pregnancies; I think basically those are some of the people that I think may not, or may be excluded from carrying out the test…most women who have cerclage, you don’t want to tamper with the cervix. Those are just some of the ones I think probably we just exclude from the study.” (KII 3, O&G)

## Discussion

In this qualitative study of pregnant women and their HCP in Nigeria, most participants, irrespective of gravidity, religious faith, and presence of other chronic health conditions such as HIV, indicated a favorable attitude toward the introduction of HPV DNA based cervical cancer screening into routine ANC. While safety of the sampling procedure during pregnancy was a concern for many pregnant women and a few midwives, there was widespread faith in recommendations made by HCP, with many participants believing that their HCP would not recommend harmful practices. Mandatory screening policy with opt-out options, affordability, or subsidization of cost was identified as important factors in increasing uptake, while lack of awareness and non-availability of screening centers were identified as major deterrents.

A woman’s decision to participate in cervical cancer screening during the antenatal period is guided by a complex interplay of individual and socio-cultural factors. A rich and comprehensive understanding of these factors is important for identification of specific attitudes or beliefs that can be targeted for change in scaling up cervical cancer screening within ANC. In our study, pregnant women identified HCP’s recommendation and mandatory antenatal screening policies as the strongest motivations for participation in HPV DNA testing for cervical cancer during routine ANC. This is consistent with several reports that suggest that provider recommendation or referral is one of the strongest predictors of initiation and maintenance of regular screening, especially for women who have never been screened (35, 36). The women reported that their strong motivation to comply with recommendations made by HCP is due to their faith in the knowledge of the HCP. Studies have also shown that it is important for health care workers to communicate effectively with patients in order to allay their fears and enable them to make informed decision (37, 38). This suggests that educating HCP and ensuring that they provide proper information to women would be critical in implementing HPV DNA-based cervical cancer screening in the antenatal period.

While the implementation of mandatory opt-out HPV DNA test-based cervical cancer screening policies in ANC may overcome several barriers and improve uptake of cervical cancer screening, it may also have significant untoward consequences, such as a decline in the uptake of antenatal services, particularly in rural areas where there is poor knowledge of the benefits of cervical cancer screening. A qualitative study evaluating the mandatory opt-out HIV testing policies in ANC centers in rural Malawi revealed that pregnant women began to avoid government hospitals for antenatal services in order to avoid the mandatory HIV testing requirement (39). However, this has not been the experience with HIV testing in Nigeria. Furthermore, mandatory screening for cervical cancer may be perceived differently and engender different responses from women compared to HIV testing. Nevertheless, careful monitoring of attendance at ANC after mandatory HPV DNA test-based cervical cancer screening will be required in order to ensure that the risks do not outweigh the benefits.

The pregnant women also identified the opinion and experiences of their pregnant counterparts who have undergone the procedure as a determinant for participating in HPV DNA test-based cervical cancer screening in ANC. Our participants reported that good experiences from other women will cause them to have a favorable attitude toward HPV DNA-based cervical cancer screening during ANC. This is similar to findings from a study in a Somali community in Minnesota where some women reported that they would not follow the health care professionals’ instructions on cervical cancer screening without asking other women in the community if they should do so (40). Our findings suggest that plans for implementation of HPV DNA test-based cervical cancer screening should engage pregnant women who would champion the innovation in their communities.

Safety of obtaining exfoliated cervical cells required for HPV DNA test was a concern for many pregnant women and some midwives. Although our study was not designed to evaluate safety of the sampling procedure, a previous study that evaluated the use of HPV DNA-based testing in pregnancy as a screening strategy to increase coverage among underscreened women in France did not report adverse events associated with the sampling procedure (17). Furthermore, Pap smears, which use similar sampling procedures, are recommended at the first antenatal visit, if indicated as per current screening guidelines in the US (41, 42). Despite an absence of adverse events associated with the sampling procedure, there are potential harms associated with the detection of transient HPV infections such as anxiety over a positive HPV test, stigmatization, discomfort from additional tests required for triage, and complications due to treatment of lesions detected (42). However, these potential harms are not unique to the use of HPV DNA-based cervical cancer screening in pregnancy. With more than 60% of pregnant Nigerian women aged 20–49 years attending at least one ANC visit during their pregnancy (20–22) and the overlap between their age and recommended age group for cervical cancer screening (42, 43), incorporating HPV DNA-based cervical cancer screening into routine ANC may be an innovative and acceptable approach to increasing screening coverage among women of child bearing age.

There remain several technical and logistics challenges to be overcome before HPV DNA-based cervical cancer screening can be effectively implemented in ANC. The advent of rapid HPV tests reduces some of the challenges associated with availability of results in the ANC setting, and this has implications for treatment of positive lesions and follow-up (44). Other issues include triage of women who are positive for high-risk HPV, timing of treatment of detected lesions, clinical validation, and calibration of HPV test kits for use during pregnancy in order to reduce the risk of overtreatment due to higher prevalence of HPV infection during pregnancy (45–47).

Our study has several limitations. Although we purposively sampled women with different religious faiths, nature of dwelling, presence of co-morbidities, and gravidity to ensure a higher degree of generalizability of our findings, we recognize that in a highly diverse population as exists in Nigeria, with multiple beliefs and cultures, our results are not necessarily generalizable to the entire population of pregnant women and HCP in Nigeria. While our study was focused on the evaluation of the attitude toward the use of HPV DNA-based testing for cervical cancer screening as part of routine ANC service in Nigeria, it is possible that our participants did not make any distinction between the use of HPV DNA based testing and other cervical cancer screening methods. Therefore, our findings may reflect the overall attitude toward the incorporation of cervical cancer screening in general into routine ANC.

The themes and subthemes identified need to be studied further in large population-based surveys. Given that the facilitators of the FGDs were highly skilled nurses/research scientists affiliated with a reputable organization in Nigeria, their positionality and the inherent power dynamic in the relationship between them and the FGD participants may have resulted in deference effects with participants providing responses that they think the facilitators would like to hear. We minimized this by including trust gaining activities in the introductory segment of the FGDs.

## Conclusion

As one of the first studies to provide contextual information on the factors that influence the acceptability of HPV DNA-based cervical cancer screening in routine ANC in a LMIC country, our study makes a valuable contribution to increasing cervical cancer screening coverage. Our findings have important implications for policy and future research on increasing cervical cancer screening coverage in underscreened populations. In this study, we examined perspectives from both HCP and recipients using a combination of in-depth interviews and FGD. The relatively large sample size of our study also enabled us to explore emerging themes till the point of data saturation and redundancy, further ensuring reliability of our findings.

We provide qualitative evidence that the incorporation of HPV DNA-based screening methods for cervical cancer into routine ANC is an acceptable approach to increasing coverage of cervical cancer screening programs in developing countries. Future research is required to investigate the feasibility of implementing this approach, as well as the triage, management, and follow-up for women who screen positive.

## Ethics Statement

The study was approved by the National Health Research Ethics Committee of Nigeria (NHREC), and we obtained written informed consent from all participants.

## Author Contributions

TEF and ED contributed equally to the concept and design of the study, data acquisition, analysis, interpretation, and drafting of the manuscript. TO and TAF contributed to data acquisition, analysis, and interpretation. AA contributed to data collection and provided critical revisions to the manuscript. CA conceived the study and study design, obtained funds, guided all aspects of the study, and provided critical revisions for intellectual content. All authors read and approved the final manuscript.

## Conflict of Interest Statement

The authors declare that the research was conducted in the absence of any commercial or financial relationships that could be construed as a potential conflict of interest. The reviewer, KF, and the handling editor declared their shared affiliation, and the handling editor states that the process nevertheless met the standards of a fair and objective review.

## References

[1] Ferlay J Shin H-R Bray F Forman D Mathers C Parkin DM. Estimates of worldwide burden of cancer in 2008: GLOBOCAN 2008. Int J Cancer (2010) 127(12):2893–917. 10.1002/ijc.25516 21351269

[2] Jedy-Agba E Curado MP Ogunbiyi O Oga E Fabowale T Igbinoba F Cancer incidence in Nigeria: a report from population-based cancer registries. Cancer Epidemiol (2012) 36(5):1–17. 10.1016/j.canep.2012.04.007.Cancer 22621842 PMC3438369

[3] ICO HPV Information Centre. Human Papillomavirus and Related Diseases Report WORLD Copyright and Permissions. (2017). Available from: http://www.hpvcentre.net/statistics/reports/XWX.pdf

[4] Vaccarella S Franceschi S Engholm G Lönnberg S Khan S Bray F. 50 years of screening in the Nordic countries: quantifying the effects on cervical cancer incidence. Br J Cancer (2014) 111:965–9. 10.1038/bjc.2014.362 24992581 PMC4150271

[5] Gakidou E Nordhagen S Ziad O Coverage of cervical cancer screening in 57 countries: low average levels and large inequalities. PLoS Med (2008) 5(6):e132 10.1371/journal.pmed.0050132 18563963 PMC2429949

[6] Bruni L Barrionuevo-Rosas L Albero G Human Papillomavirus and Related Diseases in Nigeria. Summary Report 7 October 2016. (2016). Available from: www.hpvcentre.net

[7] Denny L de Sanjose S Mutebi M Anderson BO Kim J Jeronimo J Interventions to close the divide for women with breast and cervical cancer between low-income and middle-income countries and high-income countries. Lancet (2016) 389:861–70. 10.1016/S0140-6736(16)31795-0 27814963

[8] Sankaranarayanan R Nene BM Shastri SS Jayant K Muwonge R Budukh AM HPV screening for cervical cancer in rural India. N Engl J Med (2009) 36014360(14):1385–94. 10.1056/NEJMoa0808516 19339719

[9] Bansil P Wittet S Lim JL Winkler JL Paul P Jeronimo J. Acceptability of self-collection sampling for HPV-DNA testing in low-resource settings: a mixed methods approach. BMC Public Health (2014) 14:596. 10.1186/1471-2458-14-596 24927941 PMC4061776

[10] Ndayisaba G Verwijs MC van Eeckhoudt S Gasarabwe A Hardy L Borgdorff H Feasibility and acceptability of a novel cervicovaginal lavage self-sampling device among women in Kigali, Rwanda. Sex Transm Dis (2013) 40(7):552–5. 10.1097/OLQ.0b013e31828e5aa5 23965769

[11] Bansil P Lim J Byamugisha J Kumakech E Nakisige C Jeronimo JA. Performance of cervical cancer screening techniques in HIV-infected women in Uganda. J Low Genit Tract Dis (2015) 19(3):215–9. 10.1097/LGT.0000000000000090 25551591

[12] Sellors JW Lorincz AT Mahony JB Mielzynska I Lytwyn A Roth P Comparison of self-collected vaginal, vulvar and urine samples with physician-collected cervical samples for human papillomavirus testing to detect high-grade squamous intraepithelial lesions. CMAJ (2000) 163(5):513–8. 11006761 PMC80457

[13] Chan PKS Picconi MA Cheung TH Giovannelli L Park JS. Laboratory and clinical aspects of human papillomavirus testing. Crit Rev Clin Lab Sci (2012) 49(4):117–36. 10.3109/10408363.2012.707174 22913405 PMC3469219

[14] Schiffman M Wentzensen N Wacholder S Kinney W Gage JC Castle PE. Human papillomavirus testing in the prevention of cervical cancer. J Natl Cancer Inst (2011) 103(5):368–83. 10.1093/jnci/djq562 21282563 PMC3046952

[15] Bulk S Bulkmans NW Berkhof J Rozendaal L Boeke AJ Verheijen RH Risk of high-grade cervical intra-epithelial neoplasia based on cytology and high-risk HPV testing at baseline and at 6-months. Int J Cancer (2007) 121(2):361–7. 10.1002/ijc.22677 17354241

[16] Naucler P Ryd W Törnberg S Strand A Wadell G Elfgren K Human papillomavirus and papanicolaou tests to screen for cervical cancer. N Engl J Med (2007) 357(16):1589–97. 10.1056/NEJMoa073204 17942872

[17] Brun-Micaleff E Coffy A Rey V Didelot MN Combecal J Doutre S Cervical cancer screening by cytology and human papillomavirus testing during pregnancy in French women with poor adhesion to regular cervical screening. J Med Virol (2014) 86:536–45. 10.1002/jmv 24114972

[18] Khanuja E Ghosh UK Garg P Tomar G Madan M Bansal R. A study of cervical intraepithelial neoplasia in pregnancy. J Obstet Gynaecol India (2014) 64(3):193–6. 10.1007/s13224-013-0499-7 24966504 PMC4061339

[19] Skoczynski M Gofdzicka-Józefiak A Kwavniewska A. The prevalence of human papillomavirus between the neonates and their mothers. Biomed Res Int (2015) 2015(126417):1–6. 10.1155/2015/126417 26713313 PMC4680044

[20] Osungbade K Oginni S Olumide A Content of antenatal care services in secondary health care facilities in Nigeria: implication for quality of maternal health care. Int J Qual Health Care (2009) 20(5):346–51. 10.1093/intqhc/mzn026 18621778

[21] Conrad P Schmid G Tientrebeogo J Moses A Kirenga S Neuhann F Compliance with focused antenatal care services: do health workers in rural Burkina Faso, Uganda and Tanzania perform all ANC procedures? Trop Med Int Health (2012) 17(3):300–7. 10.1111/j.1365-3156.2011.02923.x 22151853

[22] Iyaniwura C Yussuf Q. Utilization of antenatal care and delivery services in Sagamu, South Western Nigeria. Afr J Reprod Health (2009) 13(3):111–22. 20690266

[23] Nwaeze IL Enabor OO Oluwasola TAO Aimakhu CO Perception and satisfaction with quality of antenatal care services among pregnant women at the University College Hospital, Ibadan, Nigeria. Ann Ib Postgrad Med (2013) 11(1):22–8. 25161419 PMC4111061

[24] Gupta S Yamada G Mpembeni R Frumence G Callaghan-Koru JA Stevenson R Factors associated with four or more antenatal care visits and its decline among pregnant women in Tanzania between 1999 and 2010. PLoS One (2014) 9(7):e101893. 10.1371/journal.pone.0101893 25036291 PMC4103803

[25] Lincetto O Mothebesoane-Anoh S Gomez P Munjanja S Antenatal care. Opportunities for Africa’s Newborns. WHO (2006). p. 51–62. Available from: www.who.int/pmnch/media/publications/aonsectionIII_2.pdf

[26] Mirkuzie AH Sisay MM Hinderaker SG Moland KM Mørkve O. Comparing HIV prevalence estimates from prevention of mother-to-child HIV transmission programme and the antenatal HIV surveillance in Addis Ababa. BMC Public Health (2012) 12:1113. 10.1186/1471-2458-12-1113 23267693 PMC3533689

[27] Parkpinyo N Inthasorn P Laiwejpithaya S Punnarat T. Benefits of cervical cancer screening by liquid-based cytology as part of routine antenatal assessment. Asian Pac J Cancer Prev (2016) 17(9):4457–61. 10.7314/APJCP.2016.17.9.4457 27797261

[28] Meyrelles AR Siqueira JD Santos PP Hofer CB Luiz RR Seuánez HN Bonafide, type-specific human papillomavirus persistence among HIV-positive pregnant women: predictive value for cytological abnormalities, a longitudinal cohort study. Mem Inst Oswaldo Cruz (2016) 111(2):120–7. 10.1590/0074-02760150393 26872340 PMC4750452

[29] Glanz K Rimer BK Theory at a Glance: A Guide for Health Promotion Practice. San Francisco: Heal (2005). 52 p.

[30] AJZEN IA Perceived behavioral control, self-efficacy, locus of control, and the theory of planned behavior. J Appl Soc Psychol (2002) 32(4):665–83. 10.1111/j.1559-1816.2002.tb00236.x

[31] Dareng EO Jedy-Agba E Bamisaye P Isa Modibbo F Oyeneyin LO Adewole AS Influence of spirituality and modesty on acceptance of self-sampling for cervical cancer screening. PLoS One (2015) 10(11):e0141679. 10.1371/journal.pone.0141679 26529098 PMC4631343

[32] Allen JD Pérez JE Pischke CR Tom LS Juarez A Ospino H Dimensions of religiousness and cancer screening behaviors among church-going Latinas. J Relig Health (2014) 53:190–203. 10.1007/s10943-012-9606-9 22618412 PMC3929031

[33] Trotter RT II Qualitative research sample design and sample size: resolving and unresolved issues and inferential imperatives. Prev Med (2012) 55:398–400. 10.1016/j.ypmed.2012.07.003 22800684

[34] Palys T Purposive sampling. In: Given LM, editor. The SAGE Encyclopedia of Qualitative Research Methods, (Vol. 2). SAGE: Los Angeles (2008). p. 697–8.

[35] Ackerson K Preston SD A decision theory perspective on why women do or do not decide to have cancer screening: systematic review. J Adv Nurs (2009) 65(6):1130–40. 10.1111/j.1365-2648.2009.04981.x 19374678

[36] Fylan F Screening for cervical cancer: a review of women’s attitudes, knowledge, and behaviour. Br J Gen Pract (1998) 48:1509–14. 10024713 PMC1313202

[37] Levano W Miller JW Leonard B Bellick L Crane BE Kennedy SK Public education and targeted outreach to underserved women through the national breast and cervical cancer early detection program HHS public access. Cancer (2014) 120(16):2591–6. 10.1002/cncr.28819 25099902 PMC4460797

[38] Chorley AJ Marlow LAV Forster AS Haddrell JB Waller J Experiences of cervical screening and barriers to participation in the context of an organised programme: a systematic review and thematic synthesis. Psychooncology (2016) 26:161–72. 10.1002/pon.4126 27072589 PMC5324630

[39] Angotti N Dionne KY Gaydosh L An offer you can’t refuse? Provider-initiated HIV testing in antenatal clinics in rural Malawi. Health Policy Plan (2011) 26(4):307–15. 10.1093/heapol/czq066 21047809 PMC3118912

[40] Ghebre RG Sewali B Osman S Adawe A Nguyen HT Okuyemi KS Cervical cancer: barriers to screening in the Somali community in Minnesota. J Immigr Minor Health (2015) 17(3):722–8. 10.1007/s10903-014-0080-1 25073605 PMC4312274

[41] Salani R Billingsley CC Crafton SM Cancer and pregnancy: an overview for obstetricians and gynecologists. Am J Obstet Gynecol (2014) 211:7–14. 10.1016/j.ajog.2013.12.002 24316272

[42] Saslow D Solomon D Lawson HW Killackey M Kulasingam SL Cain J American Cancer Society, American Society for Colposcopy and Cervical Pathology, and American Society for Clinical Pathology screening guidelines for the prevention and early detection of cervical cancer. CA Cancer J Clin (2012) 62(3):147–72. 10.3322/caac.21139 22422631 PMC3801360

[43] Flagg EW Datta SD Saraiya M Unger ER Peters E Cole L Population-based surveillance for cervical cancer precursors in three central cancer registries, United States 2009. Cancer Causes Control (2014) 25(5):571–81. 10.1007/s10552-014-0362-x 24578200 PMC6921482

[44] Castle PE Smith KM Davis TE Schmeler KM Ferris DG Savage AH Reliability of the Xpert HPV assay to detect high-risk human papillomavirus DNA in a colposcopy referral population. Am J Clin Pathol (2015) 143:126–33. 10.1309/AJCP4Q0NSDHWIZGU 25511151

[45] Frega A Verrone A Manzara F Conclusions: An Observational Management Based on the Use of Molecular Test and Expression of E6/E7 HPV-DNA, HPV-mRNA and Colposcopic Features in Management of CIN2/3 during Pregnancy. (2017). Available from: http://www.europeanreview.org/wp/wp-content/uploads/4236-4242-CIN23-lesions-during-pregnancy.pdf

[46] Liu P Xu L Sun Y Wang AZ. The prevalence and risk of human papillomavirus infection in pregnant women. Epidemiol Infect (2014) 142(8):1567–78. 10.1017/S0950268814000636 24667102 PMC9151227

[47] Arbyn M Steben M Highlights of the 26th international papillomavirus conference and workshops. Future Oncol (2010) 6(11):1711–24. 10.2217/fon.10.136 21142658

